# Robust Denaturation of Villin Headpiece by MoS_2_ Nanosheet: Potential Molecular Origin of the Nanotoxicity

**DOI:** 10.1038/srep28252

**Published:** 2016-06-17

**Authors:** Zonglin Gu, Zaixing Yang, Seung-gu Kang, Jerry R. Yang, Judong Luo, Ruhong Zhou

**Affiliations:** 1 School for Radiological and Interdisciplinary Sciences (RAD-X) and Collaborative Innovation Center of Radiation Medicine of Jiangsu Higher Education Institutions, Soochow University, Suzhou, 215123, China; 2 Computational Biological Center, IBM Thomas J. Watson Research Center, Yorktown Heights, NY 10598, USA; 3 Department of Radiotherapy, Changzhou Tumor Hospital, Soochow University, Changzhou, 213001, China; 4 Department of Chemistry, Columbia University, New York, NY 10027, USA

## Abstract

MoS_2_ nanosheet, a new two-dimensional transition metal dichalcogenides nanomaterial, has attracted significant attentions lately due to many potential promising biomedical applications. Meanwhile, there is also a growing concern on its biocompatibility, with little known on its interactions with various biomolecules such as proteins. In this study, we use all-atom molecular dynamics simulations to investigate the interaction of a MoS_2_ nanosheet with Villin Headpiece (HP35), a model protein widely used in protein folding studies. We find that MoS_2_ exhibits robust denaturing capability to HP35, with its secondary structures severely destroyed within hundreds of nanosecond simulations. Both aromatic and basic residues are critical for the protein anchoring onto MoS_2_ surface, which then triggers the successive protein unfolding process. The main driving force behind the adsorption process is the dispersion interaction between protein and MoS_2_ monolayer. Moreover, water molecules at the interface between some key hydrophobic residues (e.g. Trp-64) and MoS_2_ surface also help to accelerate the process driven by nanoscale drying, which provides a strong hydrophobic force. These findings might have shed new light on the potential nanotoxicity of MoS_2_ to proteins with atomic details, which should be helpful in guiding future biomedical applications of MoS_2_ with its nanotoxicity mitigated.

The most common two-dimensional (2D) nanomaterials are probably those carbon-based ones, such as graphene, graphyne and their derivatives, which have attracted tremendous interests in many fields including biomedicine since its discovery^1^,^2^,^3^,^4^,^5^,^6^,^7^. Novel 2D nanomaterials, such as MoS_2_^8^, WS_2_^9^, and WO_3_^10^, are quickly catching up and emerge as a new research frontier. These materials have been featured with unparalleled structural amenability, exceptionally high specific surface area^11^, unusual size-dependent effects^12^, and excellent mechanical and electrical properties^13^,^14^. Therefore, there have been many attempts to take advantage of various 2D nanomaterials as delivery platforms, diagnostic agents, therapeutic nanodrugs, and tissue engineering scaffolds^15^,^16^,^17^,^18^,^19^,^20^,^21^.

Accompanying with these promising biomedical applications, there is also a growing concern on the biosafety and cytotoxicity of these 2D nanomaterials^22^,^23^,^24^,^25^. Taking graphene as an example, its potential cytotoxicity has been raised widely in literature. Its adverse effects on tissues, cells and various biomolecules have been heavily investigated with various experimental techniques^26^. Meanwhile, recent theoretical studies have also revealed graphene’s (including graphene oxide) influence on the integrity of cell membranes^27^,^28^,^29^,^30^, as well as protein structures^23^,^25^. Based on these findings from both experiment and theory on the fundamental mechanisms, various strategies have been developed to enhance graphene’s biocompatibility, such as functionalization with organic molecules, lipids, polymers, peptides and proteins^31^,^32^,^33^,^34^,^35^,^36^.

More interestingly, MoS_2_ (molybdenum disulfide)^8^, a branch in 2D transition metal dichalcogenides nanomaterials, is receiving a significant amount of attention lately. It is believed that molybdenum disulfide might share similar physicochemical properties with graphene, and can potentially replicate graphene’s success in biomedical applications. Recent studies indicate that it has strong antimicrobial and antifungal activity^37^,^38^. Meanwhile, a field effect biosensor has been proposed for tumor marker proteins using its unique direct band gap^39^. In addition, its high near-infrared (NIR) absorbance and extensive specific surface area makes it ideal as a novel photothermal-triggered drug delivery platform^40^. It is also proposed for cancer therapy through a combined approach with both photothermal and chemotherapeutic agents^39^,^41^. MoS_2_ can also be used as a contrast agent in X-ray tomography imaging with Mo’s excellent absorption ability^40^.

Despite these efforts, the detailed molecular interactions between MoS_2_ nanosheets and various biomolecules such as proteins remain largely unknown. In this work, we conduct all-atom molecular dynamics (MD) simulations to study the interaction of Villin Headpiece (HP35), a widely used model protein in folding studies, with MoS_2_. It is shown that MoS_2_ exhibits exceptionally robust denaturation capability to HP35, with the protein secondary structures all severely destroyed within a few hundred nanosecond simulations, indicating a potentially severe nanotoxicity. Both the aromatic and basic residues contribute to the initial protein anchoring on the surface of MoS_2_, which then trigger the successive protein unfolding process, with hydrophobic residues play a key role in the denaturing process.

## Models and Methods

The HP35 is a protein that folds independently into a three-helix bundle, which has been widely used as a model scaffold in protein folding studies. The initial protein structure used in this study was downloaded from the Protein Data Bank with the PDB code 1YRF^42^. The simulation system consisted of a HP35 protein, and a MoS_2_ nanosheet with a size of 6.735 nm × 6.600 nm. The MoS_2_ nanosheet parameters were adopted from a previous study (see Table S1 for details)^43^. The initial distance between the protein and the MoS_2_ nanosheet was set to be 0.8 nm. The complex system was then solvated in a rectangular water box (6.924 nm × 6.744 nm × 5.000 nm), containing 6,568 water molecules (21,728 atoms in total). Two chloride ions were also added to this water box to neutralize the system. This fully solvated complex was then simulated with molecular dynamics simulations, which are widely used in the studies of biomolecules^44^,^45^,^46^,^47^,^48^,^49^,^50^,^51^,^52^ and nanomaterials^23^,^53^,^54^,^55^,^56^,^57^.

The MD simulation was performed with the software package GROMACS (version 4.6.6)^58^. The VMD software^59^ was used to analyze and visualize the simulation results. We adopted CHARMM 27 force field^60^ and TIP3P water model^61^ for the protein and water molecules, respectively. Temperature was fixed at 300 K using *v*-rescale thermostat and the volume of the simulation box also remained constant during the simulation (NVT)^62^. Periodic boundary conditions were applied in all directions. To avoid the “artificial collapsing” of nanosheets with their mirror images due to the limited size of simulation box (which is due to the limited computational resource), the MoS_2_ nanosheet was frozen throughout the simulation process. The long-range electrostatic interactions were treated with PME method^63^, and the van der Waals (vdW) interactions were calculated with a cutoff distance of 1.0 nm. All solute bonds were maintained constant at their equilibrium values with the LINCS algorithm^64^, and water geometry was also constrained using the SETTLE algorithm^65^. During the production runs, a time step of 2.0 fs was used, and data were collected every 20 ps. The total aggregated simulation time was larger than 2 μs.

## Results and Discussions

**Figure 1:**
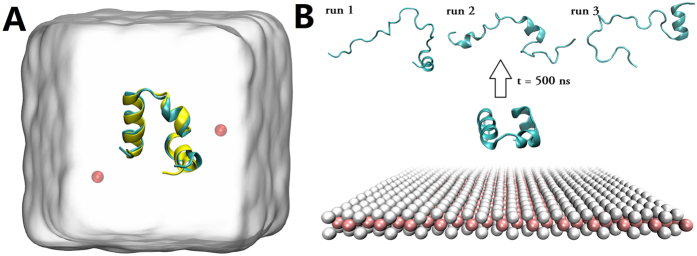
Simulations in bulk water and on MoS_2_ surface. (**A**) A snapshot of the control simulation in the bulk water. The initial and final structures of HP35, respectively marked in yellow and cyan, are superimposed in order to show their structural consistency. Water box is depicted with white surface, and two Cl^−^ ions (red spheres) are introduced to neutralize the system. (**B**) Characteristic structures of HP35 on MoS_2_ surface. The initial fold of HP35 on MoS_2_ surface is shown in the bottom panel (see Figure S1 for more view angles), and the final denatured proteins obtained at t = 500 ns from three independent runs are displayed in the top. Mo and S atoms of MoS_2_ surface are depicted with pink and white van der Waals balls, respectively.

Our simulation shows that the MoS_2_ nanosheet can be highly detrimental to the native protein folds. In all simulations with protein HP35, we found that the characteristic helical structures of HP35 have been severely destroyed on MoS_2_ surface. As featured in the final snapshots from three independent runs at 500 ns (Fig. 1B), HP35 lost most of its native *α*-helical content (~80%). This is in high contrast with the control run with protein HP35 in bulk water without the MoS_2_ nanosheet (Fig. 1A), which displays only marginal difference from the initial crystal structure.

For a more quantitative measure on how the adsorption affects the protein tertiary structure, we then monitored the atomic contacts of HP35 on the MoS_2_ surface and the native contacts of the protein. Here, a contact with MoS_2_ surface was counted when any heavy atom of the sidechain of HP35 is within 0.6 nm of any atom of MoS_2_. With a same criterion on the cut-off distance, the fraction of native contacts of protein at time *t*, *Q*(*t*), was defined as the ratio of the total number of native contacts at time *t* to that at time zero (i.e., the total number of native contacts in the x-ray crystal structure), where only residue pairs apart at least 3 consecutive residues from each other were considered.

**Figure 2:**
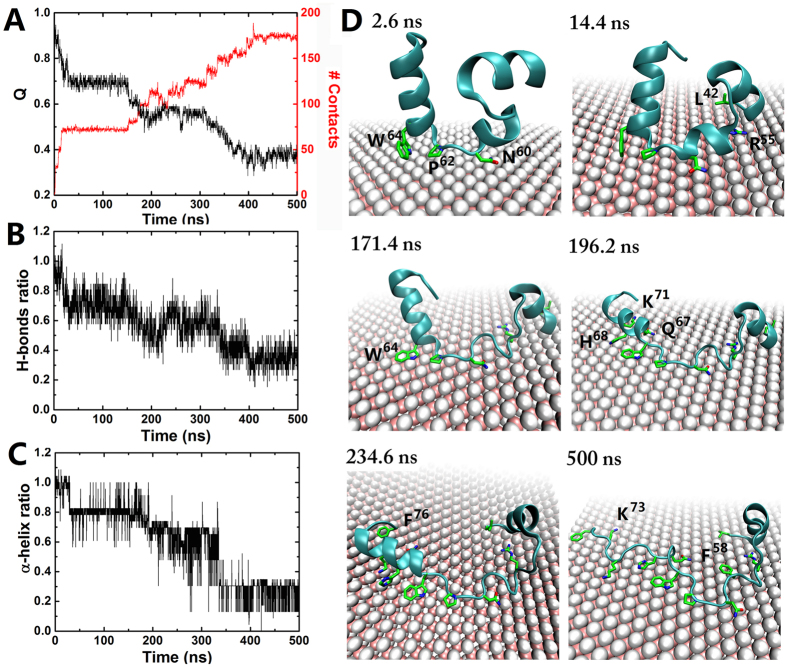
Structural dynamics of HP35 on MoS_2_ surface. (**A**) Time profile of native contact Q of HP35 (black) and heavy atom contact number between HP35 sidechains and MoS_2_ (red). (**B**,**C**) Time profiles for hydrogen bond and α-helix ratios of HP35 on MoS_2_, respectively. (**D**) Key intermediate structures of HP35 along the representative simulation trajectory. Some key residues are highlighted with sticks marked in green and blue for carbons and nitrogens, respectively. Other representation schemes are same as those used in Fig. 1.

As shown in Fig. 2, HP35 seriously loses its native contact as the adsorption progresses. Especially, the extent of protein denaturation is directly proportional to that of surface adsorption (Fig. 2A), which strongly implies that MoS_2_ directly impacts on the protein tertiary structure. Accordingly, it is accompanied with deformation on the secondary structures as well as the local hydrogen bonding network (Fig. 2B,C). More specifically, for the first 15 ns after the onset, HP35 quickly lost its native contacts by about 30%, which was mainly led by the initial contacts from residues between the second and third helices (e.g., Asn60, Leu-61, Pro62 and Trp64; see Fig. 2D at 2.6 and 14.4 ns) with an end-on orientation (rather than side-on) (see Fig. 2D at t = 2.6 ns; this pattern was also found in the other two trajectories (Figure S2)). After about 135 ns of a metastable state from 15 to 150 ns, HP35 started further denaturation again until it reached a more stable state at ~410 ns. The loss of the protein native contacts and the increase of the contacts with MoS_2_ both display a stepwise manner (Fig. 2A), indicating a fragment- or residue-based rupture of the protein structure onto the MoS_2_ nanosheet (see Fig. 2C,D).

To further understand the initial binding dynamics and underlying mechanism, the time evolution of secondary structures and heavy atom contact numbers between each residue with MoS_2_ for all the three trajectories have been analyzed in detail (Figure S3). First, the detailed kinetics for each helix unfolding does differ somewhat from trajectory to trajectory (Figure S3, first column). That said, the protein was initially separated reasonably far away from the MoS_2_ nanosheet, but some of the major events and typical conformations still correlate quite well between different trajectories. For example, both run-1 and run-3 share very similar unfolding dynamics (Figure S3) and final configurations (Fig. 1B), with the second helix firstly unfold, followed by the third helix, while the first helix remained mostly intact till the end of the simulation. Also as shown in the second column of Figure S3, for all the three simulated trajectories, the second helix (from amino acid (AA) 54 to 61) absorbed onto the MoS_2_ surface first, followed by the first helix (AA 43 to 52), and then the third helix (AA 62 to 74).

Detailed analysis on the heavy atom contact number between each residue with MoS_2_ as the function of simulation time also reveal that the first contacting (driving) residues for the second α-helix are Arg-55, Phe-58, Asn-60, Leu-61, Leu-63, and Trp-64, with the hydrophobic (including aromatic) residue account for 67%, which is larger than its “intrinsic” proportion in the second α-helix (~57%, see Table S2), indicating hydrophobic residues are preferred in general during the adsorption process. Furthermore, the second α-helix contains the highest percentage of hydrophobic residues, followed by the first α-helix, then the third α-helix, which is also consistent with the above analysis that the hydrophobic residues have played a significant role in the early adsorption process.

**Figure 3:**
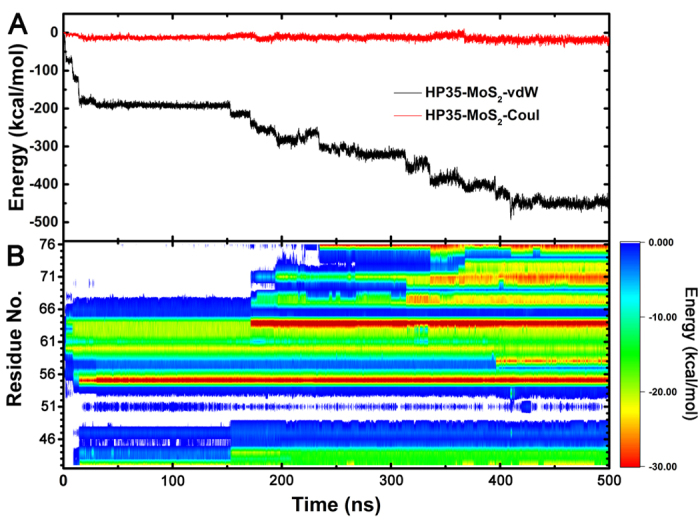
Energy dynamics of HP35 adsorption on MoS_2_ surface. (**A**) Time profiles for vdW (black) and electrostastic (red) interaction energies between HP35 and MoS_2_. (**B)** Time evolution of residue-specific vdW interaction energies of HP35 on MoS_2_ nanosheet.

To better understand the energetics responsible for the above process, we computed nonbonding interaction energies between HP35 and MoS_2_ during adsorption process. As shown in Fig. 3A, the time-profile clearly shows that the protein favors binding onto the MoS_2_ surface in terms of both electrostatic and dispersive van der Waals interaction energies. Although both interactions contribute to the binding energy as the atomic contacts with MoS_2_ increase, HP35 adsorption is mainly driven by the strong dispersion interaction between the protein and MoS_2_ surface (with the vdW interaction contributing 449.39 ± 7.79 kcal/mol, and the electrostatic interaction 19.01 ± 5.16 kcal/mol).

We further quantified the time-profile of residue-specific vdW interaction energies of HP35 with MoS_2_ nanosheet (Fig. 3B). As shown in Fig. 2D, the very initial contacts occurred at t = 2 ns with residues Asn-60, Pro-62 and Trp-64, by which the second (Asn-60 and Pro-62) and third (i.e., Trp-64) α-helices then anchored onto the MoS_2_ surface. These events induced the contacts of neighboring Leu-63 and Arg-55 at t = 9 ns. Once Arg-55 was fully settled on the surface at t = 15 ns, the first α-helix also got anchored *via* Leu-42. This state lasted for the next ~150 ns (i.e., metastable state) until several N-terminal residues started to be in contact as well. After Trp-64 was further stabilized at t = 171 ns *via* a large conformation change in its indole sidechain, the adsorption began to propagate towards the C-terminus for the next 60 ns, which drew the whole third α-helix to lay down on the surface. Once the C-terminal Phe-76 maximized its surface interaction at t = 235 ns, no additional residue made contacts on the surface but the local adjustment continued until the system found its energy minimum at t = 410 ns where the C-terminal helix was fully denatured.

**Figure 4:**
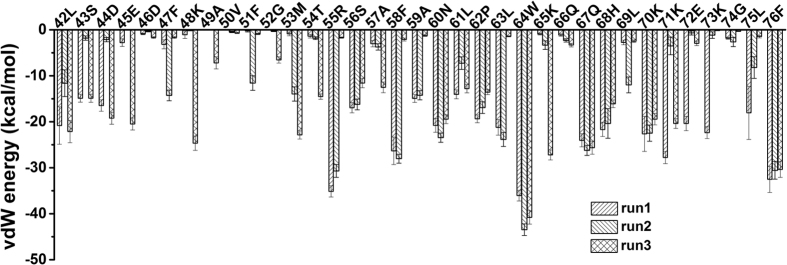
Residue-specific vdW energy profile. Dispersion energies for each residue of HP35 on MoS_2_ were averaged over the last 100 ns for each simulation trajectory.

As indicated in analyses above that the vdW interactions dominate the adsorption energetics, we then further calculated the vdW energy contributions for each residue by averaging the last 100 ns over all three independent trajectories (Fig. 4). As shown in Fig. 4, most of the highly favorable residues, with its vdW energy <−20 kcal/mol, coincide with the aforementioned residues that are important in guiding the HP35 adsorption onto MoS_2_ surface, such as Arg-55, Phe-58, Trp-64, Lys-70, Lys-71 and Phe-76. Since MoS_2_ surface is largely hydrophobic with a water contact angle around 82° 66, it is not surprising that hydrophobic residues like Phe-58, Trp-64 and Phe-76 are found in this group. However, it is interesting to note that basic residues like Arg-55, Lys-70 and Lys-71 are also in the same group. Actually, they played a crucial role in tethering the protein onto the surface of MoS_2_. For example, Arg-55 was holding the second α-helix to the surface until it is fully denatured. This important role of basic residues such as Arg is consistent with the similar role they play in their interactions with graphene and carbon nanotubes^67^, where even without any electrostatic interactions the dispersion interactions with the solvent-exposed long aliphatic sidechains in Arg and Lys contribute favorably (of course, the π–stacking between the guanidinium group and aromatic rings also contributes in the Arg case there^67^).

To probe the role of interfacial water in mediating the adsorption process, some intermediate configurations in a representative trajectory are displayed in Figure S4. For the clarity MoS_2_ was not shown. At t = 5 ns, a small, partial, drying zone was formed as marked with the black circle, between the hydrophobic region (white region) and the MoS_2_ surface. The drying zone expanded to a larger area after ~50 ns. Meanwhile, there were also some water molecules bridging the partially positive-charged region (blue region) of the protein and the surface of MoS_2_ (marked with red circle; near the drying region). At t = 235 ns, the drying region spread to the entire “MoS_2_-contacted hydrophobic region”, accompanied by a much larger overall contacting surface. Interestingly, water molecules that reside at the interval between positively charged regions of protein and MoS_2_’s surface (highlighted by the red circle), can persist throughout the entire simulation length, which implies that water may act as lubricant for the binding of these hydrophilic regions to MoS_2_. Taken together, water indeed played a significant role in the binding process: (*i*) partial dewetting occurred at the interface between the hydrophobic region of protein and the surface of MoS_2_, which provided a strong driving force for the early adsorption; and (*ii*) water acted as lubricant to facilitate adsorption of hydrophilic zone of the protein with MoS_2_.

**Figure 5:**
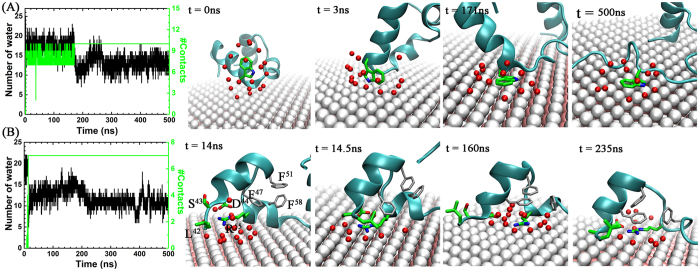
Surface adsorption dynamics for key residues, Trp-64 (**A**) and Arg-55 (**B**). Left panel: time-profiles for heavy atom contact (green curve) between the sidechain and MoS_2_, and water number in the FSS of the selected residues (black curve). Right panels: representative snapshots for key intermediate states along the adsorption process. The selected residues (64W and 55R) are depicted by sticks marked in green (carbons) and blue (nitrogens), and nearby residues (47F, 51F and 58F) by gray sticks. Water in FSS are depicted with red spheres. Other schemes are same as those used in Fig. 1.

In order to further elaborate this point on key contributors, we narrowed down our focus by selecting the two most representative residues, Trp-64 and Arg-55 (one aromatic and one basic). Since water hydration is an important factor for the surface adsorption, water population in the first solvation shell (FSS) of each residue was taken into account in addition to the time-evolution of atomic contacts on MoS_2_ surface. At the beginning, Trp-64 was solvated by 18 waters in the bulk (Fig. 5A). This number was not changed much until t = 171 ns, even after the indole ring of Trp-64 started to contact with MoS_2_ surface at t = 3 ns. In this state, the indole ring was found to vertically contact on the surface through its edge with 8 atomic contacts on average. Due to this edge (indole ring)-to-face (MoS_2_ surface) contact mode, the water population in the FSS was not noticeably affected. However, once the indole sidechain lay down on the surface at t = 171 ns, about 12 water molecules left from FSS (nanoscale drying), while the atomic contact increased by 2. The face-to-face conformation was quite stable during our simulation till 500 ns. This nanoscale drying^44^,^45^,^68^,^69^,^70^ further enhanced the binding strength of the indole ring with the MoS_2_ surface, in addition to the strong dispersive interactions.

Similarly to Trp-64, Arg-55 also displays a very stable adsorption on the MoS_2_ surface. However, the detailed process turned out to be more complicated. For the first 14 ns, Arg-55 was fully solvated with 18 waters in its FSS with no surface contact. After the onset of the adsorption, the full sidechain atoms were adsorbed within ~0.5 ns. A snapshot at t = 14.5 ns shows that guanidinium group prefers to have the face-to-face configuration toward MoS_2_ surface with a contact number of 7 (which remains the same till the end of simulation). In the meantime, about 5 water molecules were squeezed out from the interface between the residue and MoS_2_ surface until t = ~160 ns, after which the number of water in FSS slightly increased by ~2 for the next ~20 ns. The restoration was due to water intrusion to the place originally excluded by the salt-bridge formation with Asp-44 (see snapshot at t = 160 ns of Fig. 5B). Not long after the breakage, Phe-47 began to interact with Arg-55 (see snapshot at t = 235 ns of Fig. 5B), eventually forming a stable stacking *via* energetically favorable cation-π interaction^71^. By this coupling, Arg-55 became desolvated again by ~3 water molecules, meaning a total of 6 waters left from FSS of Arg-55 compared to that of the bulk.

While the hydrophobic interaction is important for the adsorption of Arg’s aliphatic chain, our analysis indicates that more complicated interactions are involved in this case with the guanidinium moiety. Due to the net positive charge, a similar dramatic nanoscale drying as seen in Trp-64 is not likely in Arg-55 (only 6 waters left from the FSS of Arg-55 as compared to 12 in Trp-64). Furthermore, the electrostatic interactions with Phe-47 *via* cation-π interaction also contributed to the stability. Overall, the stable binding of Arg-55 on MoS_2_ surface is contributed by both dispersion and electrostatic interactions.

## Conclusion

In recent years, MoS_2_ has attracted a great deal of attention in the biomedical field due to its unique properties. Applications of MoS_2_ include the near-infrared (NIR) photothermal-triggered drug delivery platform, as well as photothermal and chemotherapy combined therapeutic agents of cancer. The MoS_2_ nanosheets can be used as contrast agents in X-ray computed tomography imaging and field-effect biosensors for label free sensitive detection of cancer marker proteins in solution. Although these applications have clearly demonstrated the importance of MoS_2_ nanosheets in biomedicine, their wider applications demand more studies on their biosafety, in particular the molecular origin of their potential nanotoxicity. Furthermore, the detailed molecular interactions between MoS_2_ nanosheets and biomolecules, such as proteins and membrane lipids, are still largely unclear. In this study, we examined the adsorption process of a model protein, Villin Headpiece (HP35), onto the surface of MoS_2_ using all-atom molecular dynamics (MD) simulations. We discovered that MoS_2_ exhibits exceptionally robust denaturation capability to Villin Headpiece, which is comparable to that of graphene nanosheet, where nearly 80% of α-helix content was destroyed after 500 ns simulation^72^.

As shown in all the three simulated trajectories, the overall structure of HP35 was severely destroyed. Both aromatic residues and basic residues were shown to play a very important role in anchoring the protein onto the surface of MoS_2_, which lead to successive profound denaturation. The strong direct dispersion interactions between key residues (such as Arg-55, Asn-60, Pro-62, Leu-63, Trp-64) and MoS_2_ were identified as the main driving force for the entire adsorption process (similar to the case of graphene nanosheet^72^). Meanwhile, water molecules at the interfacial region between some key residues (e.g. Trp-64) and MoS_2_ nanosheet were also found to play an important role due to a nanoscale drying, which accelerated the adsorption process. The similar important role of water was also identified previously in mediating the adsorption of amyloid fibrils onto graphene nanosheets^73^, as well as blood proteins onto carbon nanotubes^74^ and graphene nanosheets^75^,^76^.

Further studies on the nanotoxicity of MoS_2_ to other proteins, DNA, cell membranes, as well as cells, tissues, and animal models are highly desired for a deeper understanding of its toxicity. We also envision more development efforts on applying MoS_2_ as a new type of 2D nanomaterials in biomedical applications such as antibacterial agents. These studies on MoS_2_ toxicity can also stimulate and facilitate the cytotoxicity studies of other related nanomaterials in this emerging field of nanotoxicology.

## Additional Information

**How to cite this article**: Gu, Z. *et al.* Robust Denaturation of Villin Headpiece by MoS_2_ Nanosheet: Potential Molecular Origin of the Nanotoxicity. *Sci. Rep.* **6**, 28252; doi: 10.1038/srep28252 (2016).

## Supplementary Material

Supplementary Information
