# Supplementary material for: Robust Denaturation of Villin Headpiece by MoS2 Nanosheet: Potential Molecular Origin of the Nanotoxicity
Source: Sci Rep. 2016 Jun 17;6:28252. doi: 10.1038/srep28252 (PMC4911589; doi:10.1038/srep28252)
Supplement: Supplementary Information [file srep28252-s1.pdf]

Robust Denaturation of Villin Headpiece by MoS_2 Nanosheet: Potential Molecular Origin of the Nanotoxicity

Zonglin Gu[1,‡], Zaixing Yang[1,‡], Seung-gu Kang[2], Jerry R. Yang[2], Judong Luo[*,3,1],Ruhong Zhou[*1,2,4]

1. School for Radiological and Interdisciplinary Sciences (RAD-X) and Collaborative Innovation Center of Radiation Medicine of Jiangsu Higher Education Institutions, Soochow University, Suzhou, China, 215123

2. Computational Biological Center, IBM Thomas J. Watson Research Center, Yorktown Heights, NY 10598, USA

3.Department of Radiotherapy, Changzhou Tumor Hospital, Soochow University, Changzhou, 213001, China

4. Department of Chemistry, Columbia University, New York, NY 10027, USA

‡These authors contribute equally

*Corresponding authors: (J.D.L) Judongluo@163.com; (R.H.Z.) ruhong@us.ibm.com

Table S1. Force field parameters used in the simulation. The values are in units of eV and Å for energy and distance, respectively.

Bond coefficients	r_0	k
Mo-S	2.41	4.3213

Angle coefficients	θ_0	k
Mo-S-Mo	81.67	3.4297
S-Mo-S	81.78	2.7227

Non-bonded coefficients	σ	ε	charge
Mo	2.5510	0.8382	0.76
S	3.3695	0.0606	-0.38

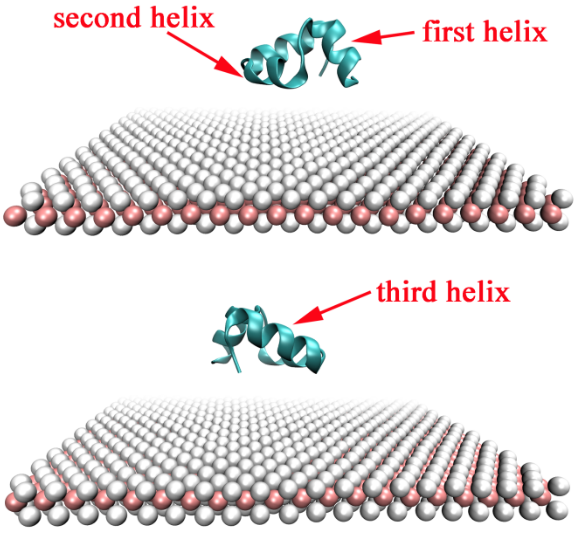

Figure S1. Two side views of HP35/MoS_2 complex.

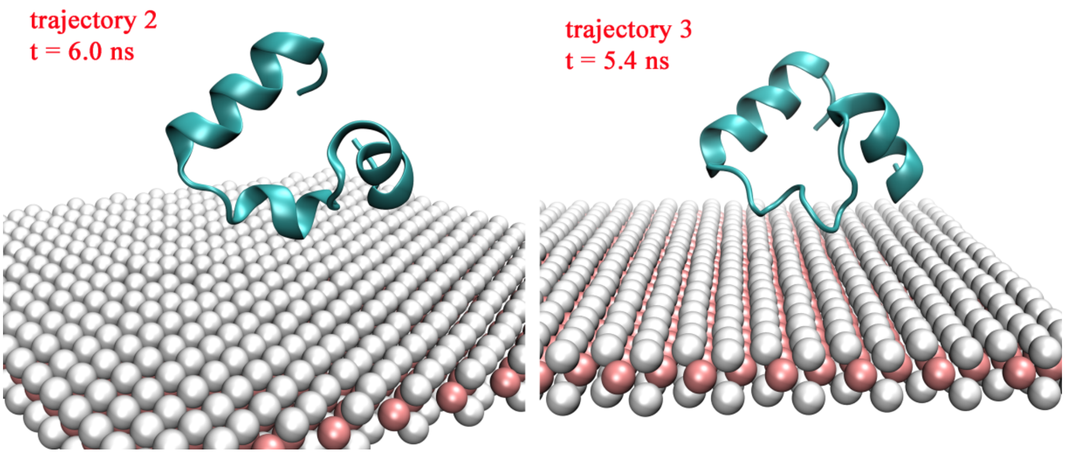

Figure S2. Initial contacting configurations of HP35 and MoS_2 nanosheet in the other two trajectories.

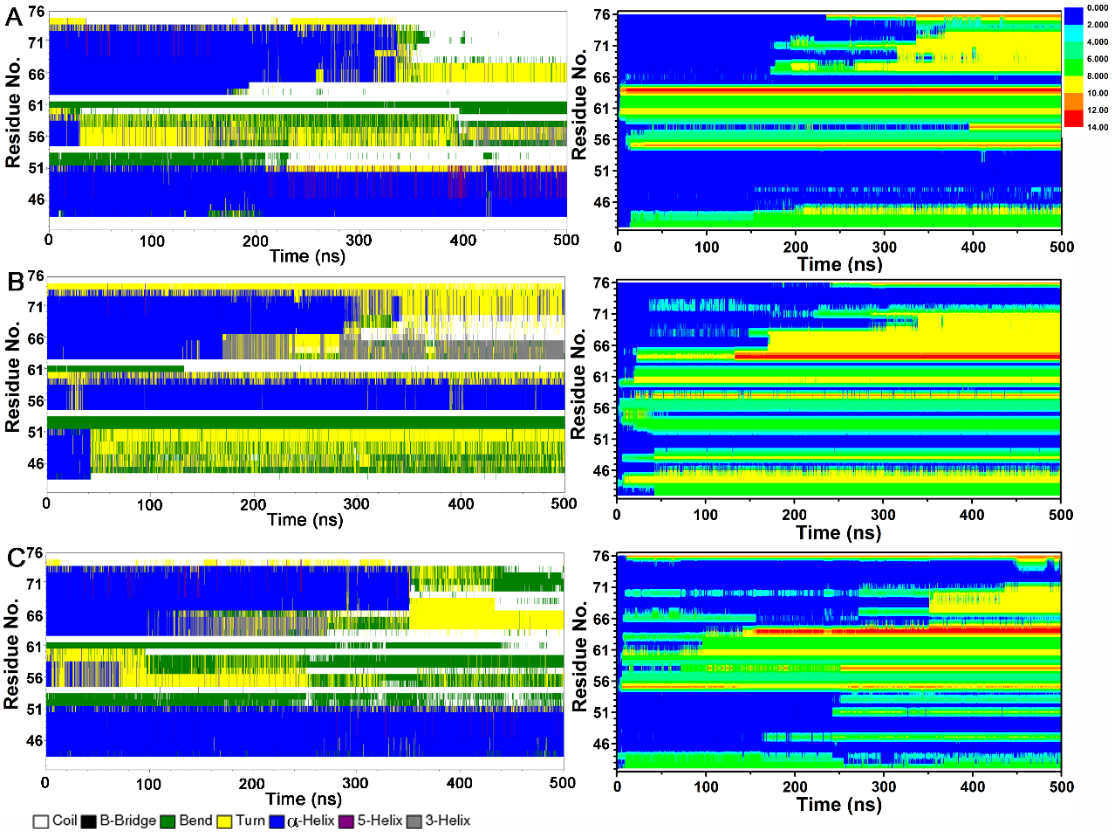

Figure. S3. Time evolution of the secondary structure of HP35 (the first column) and the heavy atom contact number between protein and MoS_2 nano-sheet (the second column) for all three trajectories, A for run-1, B for run-2, and C for run-3.

Table S2**:** Relative populations of hydrophobic, aromatic and basic residues in every helix.

	hydrophobic	aromatic	basic
First helix	30.0%	20.0%	10.0%
Second helix	42.9%	14.3%	14.3%
Third helix	23.1%	7.7%	38.5%

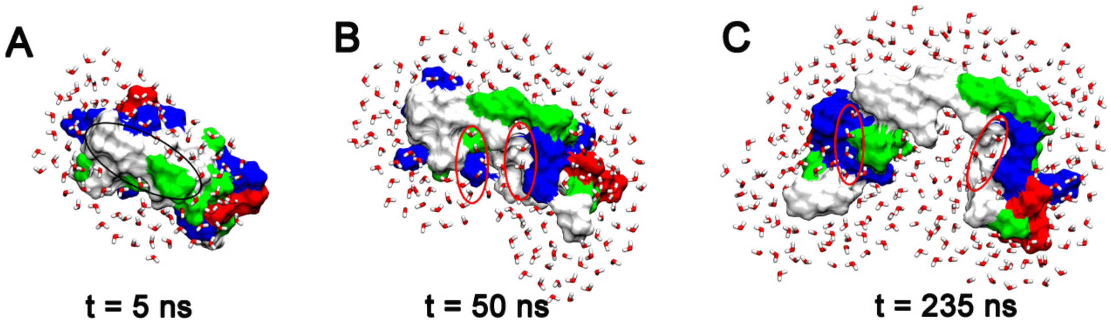

Figure S4. Representative trajectory snapshots to show the water dynamics during the adsorption of HP35 onto the surface of MoS_2 nanosheet. These important intermediate configurations are drawn from the bottom view (from the MoS_2) and hide the MoS_2 nanosheet. The HP35's surface is colored in terms of the residue sidechain's hydrophobicity, with acidic, basic, polar and nonpolar colored with red, blue, green and white, respectively. The black circle filters out the drying zone and red circles indicate the key wetting positions in which waters emerge as lubricant.
